# Optic Nerve Drusen Evaluation: A Comparison between Ultrasound and OCT

**DOI:** 10.3390/jcm11133715

**Published:** 2022-06-27

**Authors:** Nicola Rosa, Maddalena De Bernardo, Giulia Abbinante, Gianluca Vecchio, Ferdinando Cione, Luigi Capasso

**Affiliations:** 1 Department of Medicine, Surgery and Dentistry, Scuola Medica Salernitana, University of Salerno, Via S. Allende 43, 84081 Baronissi, Italy; nrosa@unisa.it (N.R.); giulia.abbinante@gmail.com (G.A.); gianluca.vecchio@hotmail.it (G.V.); nandocione1993@gmail.com (F.C.); 2 Corneal Transplant Unit, ASL Napoli 1, 80100 Naples, Italy; luigicapasso@hotmail.com

**Keywords:** B-scan, OCT, optic disc drusen, PHOMS, ultrasound

## Abstract

This observational study compared optic coherence tomography (OCT) and B-scan in the detection of optic disc drusen. In total, 86 eyes of 50 patients with optic disc drusen (ODD) (36 bilateral) with a mean age of 34.68 ± 23.81 years, and 54 eyes of 27 patients with papilledema, with a mean age of 35.42 years ± 17.47, were examined. Patients with ODD, diagnosed with ultrasound, underwent spectral-domain OCT evaluation. With US, 28 ODD cases were classified as large (4 buried and 24 superficial), 58 were classified as point-like (6 buried, 49 superficial and 3 mixed). Then, all patients underwent OCT. OCT was able to detect the presence of ODD and/or peripapillary hyperreflective ovoid mass structure (PHOMS) in 69 eyes (*p* < 0.001). In particular, 7 eyes (8.14%) showed the presence of ODD alone, 25 eyes (29.07%) showed only PHOMS and 37 eyes (43.02%) showed ODD and PHOMS. In 17 eyes (19.77%) no ODD or PHOMS were detected. In the papilledema group, no ODD were observed with both US and OCT. OCT showed the presence of drusen or similar lesions in only 80.23% of the cases highlighted by the US scan, so it does not allow for certain ODD diagnoses, especially in the case of buried ODD.

## 1. Introduction

The distal, intraocular portion of the optic nerve, known as the optic disc, can be affected by congenital and acquired pathologies. These can be diagnosed with several imaging methods, among which are ophthalmoscopic examination, optical coherence tomography (OCT) and A- and B-scan ocular ultrasound (US) [1,2,3].

The optic disc analysis is widely used to diagnose intracranial hypertension and optic neuritis, which, causing an edema of this structure, appears swelled and with blurred margins [4].

Sometimes, a pseudopapilledema can simulate an optic disc edema [5].

Pseudopapilledema can be due todifferent etiologies, among which, the most frequent are the optic disc drusen (ODD), not to be confused with the macular drusen, present in age-related macular degeneration [6].

ODD are proteinaceous materials made of calcium, mucopolysaccharides and amino nucleic acid deposits [7]. They can be superficially located in the papilla (superficial drusen) or even more deeply (deep drusen, also known as “buried drusen”), thus altering the optic disc contour.

Superficial drusen are visible with a fundus examination at the level of the optic disc and, being calcific, they can be highlighted with both autofluorescence and echography [8].

Buried drusen located in the optic disc’s deeper layers appear as non-autofluorescent amorphous masses with distinct edges. ODD, usually asymptomatic, can cause visual field defects and optic atrophy if they are large, or they can lead to choroidal neovascularization, hemorrhages and vascular occlusions. Currently, the gold standard in the ODD diagnosis is represented by US, which shows hyperreflective lesions at the optic disc level, followed by acoustic shadowing [9].

Recently, new methods, such as OCT, which seems to be able to highlight drusen, have emerged; [10] this methodology questions the role of US as the gold standard for such a purpose [11,12,13,14,15].

The purpose of the present study is to compare OCT and US reliability in the diagnosis of ODD.

## 2. Materials and Methods

### 2.1. Patients Selection

In the present study, the clinical records of patients referred to the Ophthalmic Ecographic Service of the Salerno University Hospital, from December 2018 to February 2020, with the ophthalmoscopic appearance of optic disc elevation, were reviewed to exclude the presence of papilledema. Patients with visible and/or buried drusen, unilateral or bilateral, confirmed in all cases with a B-scan ultrasound [16,17] utilizing a Cinescan S or ABSolu by Quantel Medical, Cournon-d’Auvergne, France, were examined with optical coherence tomography (OCT) (Spectralis, Heidelberg Engineering, Franklin, TN, USA) utilizing enhanced depth imaging (EDI) of the optic nerve head (8.9 × 8.9 mm, 768 × 768 pixel). The OCT scans were performed by several operators, but the images were evaluated by a single expert examiner.

Another group, composed of patients with papilledema who underwent complete neurological and ophthalmological examinations, was examined to analyze the optic nerve head in order to detect the possible presence of ODD with both OCT and US.

The study was carried out in adherence with the tenets of the World Medical Association’s Declaration of Helsinki, Institutional Review Board (IRB). Approval was obtained (Cometico Campania Sud, Italy, prot. n° 16544) and informed consent was achieved from all participants included in the study.

### 2.2. Instruments and Methods

**Figure 1:**
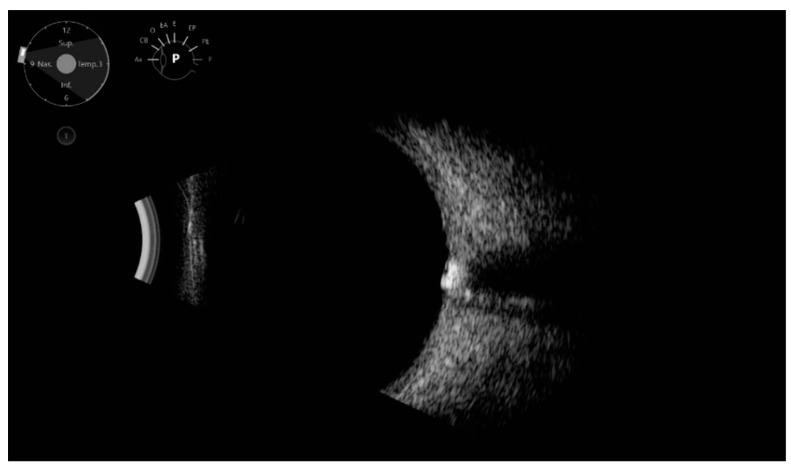
Ultrasound: Large optic disc drusen, visible as a highly reflective structure with posterior shadowing at the optic nerve level.

**Figure 2:**
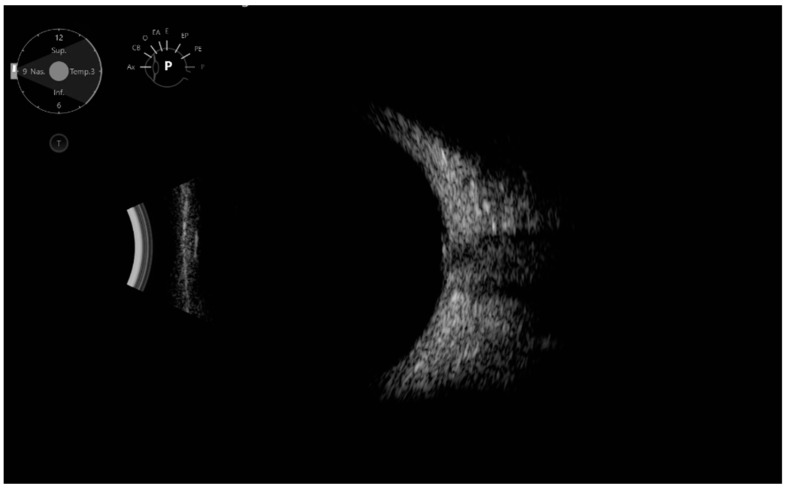
Ultrasound: Point-like optic disc drusen, visible as a highly reflective structure with a small posterior shadowing at the optic nerve level.

Ecographic ODD diagnoses were made when highly reflective structures with posterior shadowing on the optic nerve level were detected, and they were classified into large or point-like ODD (Figure 1 and Figure 2).

**Figure 3:**
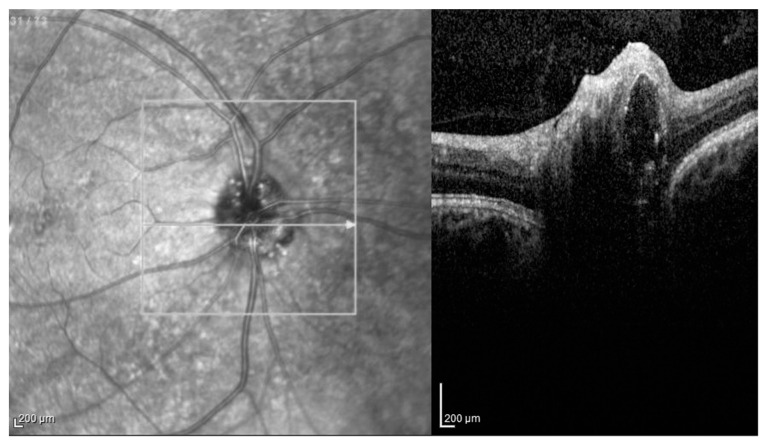
Optical coherence tomography: Large optic disc drusen, visible as a hypo-reflective structures with a total or partial hyperreflective margin, as described by the Optic Disc Drusen Studies (ODDS) Consortium. The hyperreflective margin is often more evident superiorly and can be difficult to detect.

**Figure 4:**
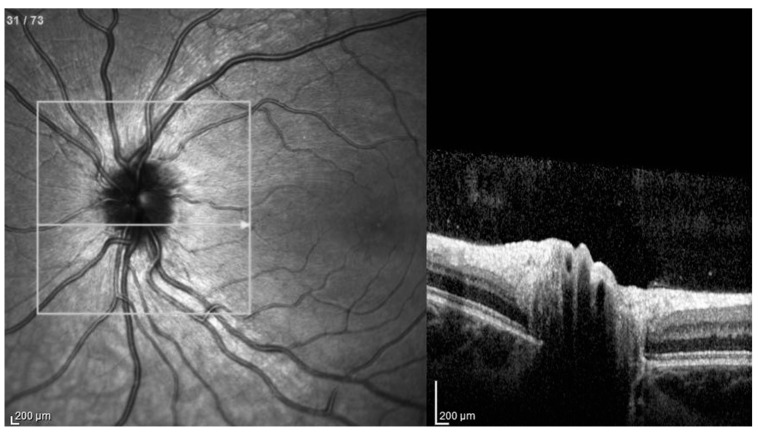
Optical coherence tomography: A hyperreflective peripapillary structure similar to an ovoid mass peripapillary hyperreflective ovoid mass structure (PHOMS), as described in patients with optic disc drusen.

The OCT images were evaluated according to the ODD Studies Consortium protocol and diagnosed as hypo-reflective structures with a total or partial hyperreflective margin (ODD) (Figure 3). In the same study, peripapillary hyperreflective ovoid mass structures (PHOMS), visible as hyperreflective peripapillary structures of an almost ovoidal shape (Figure 4), were described as possible ODD precursors or variants [16].

### 2.3. Statistical Analysis

To examine the relationship between US and OCT ODD examination, all data were analyzed with SPSS software (IBM SPSS statistics, Chicago, IL, USA, version 25) utilizing a chi-squared test and Fisher’s exact test.

The sensitivity and specificity, with 95% confidence intervals; the positive predictive value (PPV); and the negative predictive value (NPV) were analyzed, considering the B-scan ultrasound as the gold standard in ONDH diagnosis.

## 3. Results

In total, 86 eyes of 50 patients (33 women and 17 men), with ages ranging from 7 to 76 years (mean: 34.68 years ± 23.81,) diagnosed as having ODD with ocular B-scan US (Cinescan S or ABSolu by Quantel Medical), were selected. Thirty-six patients had bilateral ODD (24 women and 12 men).

Among these, 28 ODD were classified as large (4 buried and 24 superficial), and 58 were classified as point-like (6 buried, 49 superficial and 3 mixed).

If we consider PHOMS as a marker for the presence of drusen, OCT was able to detect the presence of ODD and/or PHOMS in 69 eyes out of 86 (*p* < 0.001), with a sensitivity of 80.23% and a concordance index between both tests (k) of 0.198. In particular, 7 eyes (8.14%) showed the presence of ODD alone, 25 eyes (29.07%) showed only PHOMS and 37 eyes (43.02%) showed ODD and PHOMS. In 17 eyes (19.77%) no ODD or PHOMS were detected.

If we exclude PHOMS from the evaluation, OCT was able to detect the presence of ODD in 44 eyes out of 86 (*p* < 0.001), with a sensitivity of 51.16% and a concordance index between both tests (k) of 0.488. Fisher’s exact test was used to evaluate differences between the OCT and ultrasound groups (<0.001).

Referring to drusen subcategories, by comparing the OCT findings to the ecographic ones, we obtained the following results for OCT:

Large buried: In two cases, we found both ODD and PHOMS; in two cases, OCT could not detect either ODD or PHOMS;
Large superficial: In 3 cases, we found only ODD; in 3 cases, only PHOMS; in 14 cases, both ODD and PHOMS; and in 4 cases, OCT could not detect either ODD or PHOMS;
Point-like buried: In one case, we found only ODD; in two cases, only PHOMS; in one case, both ODD and PHOMS; and in two cases, OCT could not detect either ODD or PHOMS;
Point-like superficial: In 3 cases, we found only ODD; in 20 cases, only PHOMS; in 18 cases, both ODD and PHOMS; and in 8 cases, OCT could not detect either ODD or PHOMS;
Point-like mixed superficial and buried: In two cases, we found both ODD and PHOMS, and in one case, OCT could not detect either ODD or PHOMS;

In the fellow eyes, where the US showed no ODD, OCT too was not able to detect either ODD or PHOMS.

In the papilledema group, 54 eyes of 27 patients (20 women and 7 men) with ages ranging from 8 to 78 years (mean: 35.42 years ± 17.47) were analyzed. In this group, ODD were not detected in any patient with either US or OCT.

The analysis of EDI-OCT diagnostic validity demonstrated a sensitivity of 51%, a specificity of 100%, a positive predictive value of 100% and a negative predictive value of 56.3%. Among the ODD group, 44 eyes were true positives and 42 false negatives. On the contrary, in the papilledema group, there were 54 true negatives and zero false positives.

## 4. Discussion

Over the years, US has been considered the gold standard in the detection of drusen, and for this reason, to show the reliability of other techniques, they have been compared to US by several authors [16,17]. In the case of OCT, in the international literature, the achieved results are not univocal, and they may be related to the different criteria utilized to make OCT diagnoses of ODD. In fact, some authors did not describe ODD characteristics at all, making the comparison difficult [12]. Some classified them as hypo-reflective, surrounded by hyperreflective areas [11,13,15,16,17,18,19,20], while others described them as hyperreflective [14,21]. Others described them as both hypo- and a hyperreflective, according to the localization, thus creating some confusion [22]. This discrepancy can also be seen in reviews by Silverman [13], Allegrini [15], Costello [20] and Tuğcu [23].

Mainly with US, Leon et al. [12] examined 46 children with calcific ODD. Furthermore, they claimed that since drusen are often not calcified in children, they may not be visible under B-scan, and so the preferred device should be EDI-OCT. Unfortunately, they specify that most of the patients were not evaluated with OCT, and the OCT characteristics of ODD were not described.

Among the authors that evaluated ODD as hypo-reflective regions surrounded by short hyperreflective bands, Merchant et al. [11] found, in 68 patients, that EDI-OCT could be the most effective method to evaluate ODD in children because it allows us to evaluate the ODD structure, even if performing this examination is rather difficult because of the poor collaboration of the young patients. The authors compared conventional OCT, EDI-OCT and US as diagnostic methods. EDI-OCT showed a significantly higher rate of detection of ODD than US in patients with clinically defined or suspected ODD. In no case of ODD diagnosed with US did ODD escape EDI-OCT.

Flores-Rodrıguez et al. [16] studied 66 patients with ODD, 31 patients with optic disc edema (ODE) and 70 controls to evaluate the efficacy of time-domain OCT and spectral-domain OCT in differentiating between ODD and optic disc edema (ODE). The authors analyzed both internal contour irregularities with an abrupt decrease in the hypo-reflective space and the presence of focal, hyperreflective, subretinal papillary masses as qualitative parameters of ODD presence. However, the quantitative parameters of papillary elevation and RNFL measurements showed greater sensitivity and specificity than the qualitative ones.

Gili et al. [17] analyzed 83 patients with ODD, 20 patients with pseudopapilledema (without drusen) and 37 patients with ODE in order to evaluate the efficacy of EDI-OCT in differentiating between ODD and ODE. The authors described ODD as hypo-reflective intrapapillary structures surrounded by hyperreflective horizontal lines. According to their results, EDI-OCT demonstrated a sensitivity of 92% and a specificity of 96%, highlighting that EDI-OCT allowed for differentiation between ODD and ODE.

In both of the aforementioned studies, the ODD diagnosis was obtained considering B-scan ultrasound as the gold standard in ODD diagnosis. However, they evaluated different pathologies included in the optic disc edema group, and an OCT evaluation with a one-line HD raster could miss the presence of ODD. On the contrary, the present study was focused only on ODD evaluation, and a volumetric examination of the optic nerve head (8.9 × 8.9 mm, 768 × 768 pixel) was performed.

Lee et al. [19], in 92 patients, classified ODD as focal, hyperreflective masses with an optical reflectance similar to the internal and external plexiform layers. Moreover, they described a finding that, in their opinion, could be diagnostic for the presence of drusen: a boot-shaped area near the ODD forming an acute angle between the ODD and the retinal pigment epithelium. However, no evaluation with US was performed.

Caramoy et al. [21] conducted a study on 28 patients. Among the different imaging modalities utilized, ultrasound showed the highest sensitivity (1.0). The authors concluded that superficial drusen can be evaluated by examining the fundus oculi with autofluorescence, OCT and ultrasound, while deep drusen can only be evaluated ultrasonographically.

Kulkarni et al. [22] postulated that ODD could be observed with OCT as hypo-reflective spaces owing to a shadowing effect, with anterior and posterior reflectance lines in the case of superficial ODD, while as hyperreflective masses in the case of buried ODD. The authors studied 16 eyes of nine patients with deep ODD using SD-OCT, and eight eyes of ODD patients using EDI-OCT, comparing the results obtained with 12 eyes of six patients with papilledema. Among the ODD patients, previously diagnosed with US, in several cases, they observed a hyperreflective mass with buried ODD. They concluded that SD-OCT is not reliable in differential diagnosis.

In 2018, the ODDS Consortium defined ODD characteristics [18].

To try to avoid this confusion, we decided to utilize the ODDS Consortium protocol, describing ODD as hypo-reflective structures with a total or partial hyperreflective margin. In the same study, PHOMS were described as the herniation of distended axons into the peripapillary retina [18] rather than possible ODD precursors or variants, as described by Traber et al. [24].

Unfortunately, PHOMS are also present in a variety of diseases, including papilledema, nonarteritic anterior ischemic optic neuropathy, central retinal vein occlusion, acute demyelinating optic neuritis, ODD and tilted disks [25].

For this reason, in the present study, we evaluated our results with and without the presence of PHOMS.

Jia et al. [14] did not refer to the ODDS Consortium criteria but described ODD as high-reflex dense points with different volumes of RNFL shadows in an examination of 11 adolescents with ODD and mild visual impairments using SD-OCT, US and fluorangiography. They found ODD only in six patients with US, while with OCT, they found ODD to be present in all patients.

According to this study results, therefore, with SD-OCT, buried drusen, which are not visible with ultrasound, could be found.

In conclusion, according to our results, based on the Consortium criteria, OCT does not allow for a definite ODD diagnosis, as their characteristics are often blurred, there may be artifacts and it is often difficult to distinguish ODD from the vessels themselves. Therefore, especially in case of buried ODD, the most reliable and reproducible method remains the optic nerve US. In fact, OCT revealed the presence of drusen or similar lesions only in 80.23% of the cases highlighted by the US scan.

The differential diagnosis of ODD with other congenital optic nerve anomalies or hereditary optic neuropathies, including optic nerve coloboma, optic nerve pit, myelinated nerve fibers and morning glory disc anomaly, is of fundamental importance. US, in association with a careful fundus examination, seems to be essential in the differential diagnosis [26,27,28,29,30].

This is the reason why we utilized a sample of patients with papilledema as control group and not normal subjects, as we thought that it was more important to differentiate patients with optic nerve drusen from patients with papilledema and not from patients with normal optic discs.

In summary, we can say that, so far, OCT is not a valid alternative to the ODD detection method.

## Data Availability

The datasets generated and analyzed during the current study are available from the corresponding author upon reasonable request.
